# Epidermal radio frequency electronics for wireless power transfer

**DOI:** 10.1038/micronano.2016.52

**Published:** 2016-10-24

**Authors:** Xian Huang, Yuhao Liu, Gil Woo Kong, Jung Hun Seo, Yinji Ma, Kyung-In Jang, Jonathan A. Fan, Shimin Mao, Qiwen Chen, Daizhen Li, Hank Liu, Chuxuan Wang, Dwipayan Patnaik, Limei Tian, Giovanni A. Salvatore, Xue Feng, Zhenqiang Ma, Yonggang Huang, John A. Rogers

**Affiliations:** 1 Department of Biomedical Engineering, School of Precision Instrument and Opto-electronics Engineering, Tianjin University, Tianjin 300072, China; 2 Department of Materials Science and Engineering, University of Illinois at Urbana-Champaign, Urbana, IL 61801, USA; 3 Department of Electrical and Computer Engineering, University of Wisconsin-Madison, Madison, WI 53706, USA; 4 Department of Civil and Environmental Engineering, Northwestern University, Evanston, IL 60208, USA; 5 Department of Engineering Mechanics, Center for Mechanics and Materials, Tsinghua University, Beijing 100084, China; 6 Department of Mechanical Engineering, Northwestern University, Evanston, IL 60208, USA; 7 Department of Robotics Engineering, Daegu Gyeongbuk Institute of Science and Technology, Daegu 42988, Republic of Korea; 8 Department of Electrical Engineering, Stanford University, Stanford, CA 94305, USA

**Keywords:** antenna design, epidermal electronics, modularization, silicon nanomembrane, soft-contact lamination, specific absorption rate, wireless power

## Abstract

Epidermal electronic systems feature physical properties that approximate those of the skin, to enable intimate, long-lived skin interfaces for physiological measurements, human–machine interfaces and other applications that cannot be addressed by wearable hardware that is commercially available today. A primary challenge is power supply; the physical bulk, large mass and high mechanical modulus associated with conventional battery technologies can hinder efforts to achieve epidermal characteristics, and near-field power transfer schemes offer only a limited operating distance. Here we introduce an epidermal, far-field radio frequency (RF) power harvester built using a modularized collection of ultrathin antennas, rectifiers and voltage doublers. These components, separately fabricated and tested, can be integrated together via methods involving soft contact lamination. Systematic studies of the individual components and the overall performance in various dielectric environments highlight the key operational features of these systems and strategies for their optimization. The results suggest robust capabilities for battery-free RF power, with relevance to many emerging epidermal technologies.

## Introduction

Recent research and development in wearable technologies has yielded a broad range of devices with applications in daily health monitoring^1^, activity tracking^2,3^, data logging^4^, human–machine interfaces^5,6^ and clinical diagnostics^7,8^. Future advances in this rapidly evolving area will improve processes for delivering health care^9^ and for reshaping personal lifestyles to enhance well-being^10^. A significant challenge in the creation of comfortable, non-irritating interfaces with the body originates from the current use of rigid or semi-rigid substrates and packages directly adopted from those found in non-wearable consumer electronics^1^. The result is a mechanical and geometrical mismatch with the soft and curvilinear textures of the body, thereby necessitating the use of fixtures (wrist bands, head bands, chest straps or glasses)^11^ or strong adhesives for mounting. Additional negative consequences include inconsistent, unreliable coupling to the skin, discomfort associated with contact pressure and interfacial shear and frictional forces, and constraints on locations for body integration, thereby adversely affecting the user experience and sensor accuracy. Such circumstances motivate the development of wearable devices that offer improved compatibility with the skin at the level of the materials, the geometries, the mass density, the mechanical properties and the air/water permeability^12,13^. Recent work^14–17^ has established various ultrathin, soft electronic technologies, sometimes referred to as epidermal electronic systems (EES). Such EES have physical properties that approximate those of the epidermis, integrating both passive sensing functions^18,19^ and active modalities^20^ for daily healthcare monitoring with maximum comfort and high sensing precision.

The practical feasibility of EES for continuous monitoring outside of hospital or laboratory settings rests critically on unusual schemes for the power supply because batteries^21^ and external connections^15,17,18,20^ are unacceptable. Although recent research provides solutions that include stretchable supercapacitors^22^, batteries^23^ and miniaturized platforms for harvesting energy from incident light^24^, thermal gradients^25^, mechanical motions^26^ and near-field electromagnetic (EM) waves^27,28^, additional options could be useful. Possibilities range from inductive coupling^29,30^, mid-field microwave propagation^31^ to far-field radio-frequency (RF) harvesting^32–34^, each of which exists as some forms of wireless source of power for conventional biomedical devices. The third approach is particularly attractive when implemented in commonly available industry-science-medical frequency bands owing to its versatility in power transfer and operation frequency and its long range transmission capabilities. Conventional hardware for such devices^27,34–37^ demands, however, commercial-off-the-shelf components that are challenging to render into forms suitable for chronic integration with the skin. An epidermal embodiment would, therefore, be attractive.

Here we introduce materials and integration concepts for such a technology, in which the functional components exploit ultrathin active and passive elements configured for soft, elastic mechanical properties. These wireless RF power systems use a laminated construction that facilitates optimization of analog performance, with capabilities for robust operation under significant levels of mechanical deformation. Detailed studies of the device characteristics, including the effects of RF absorption in the human body, define the key design considerations. Wireless operation of small-scale light-emitting diodes (LEDs) in full epidermal platforms mounted on the skin of the arm and placed at distances of several meters from an RF transmitter and of several centimeters from a standard cell phone, both with and without device deformation, illustrates the performance. The results establish an attractive option for wireless power delivery to epidermal systems of various types for both healthcare and non-healthcare related applications.

## Materials and methods

### Modularization approach and soft contact lamination

**Figure 1:**
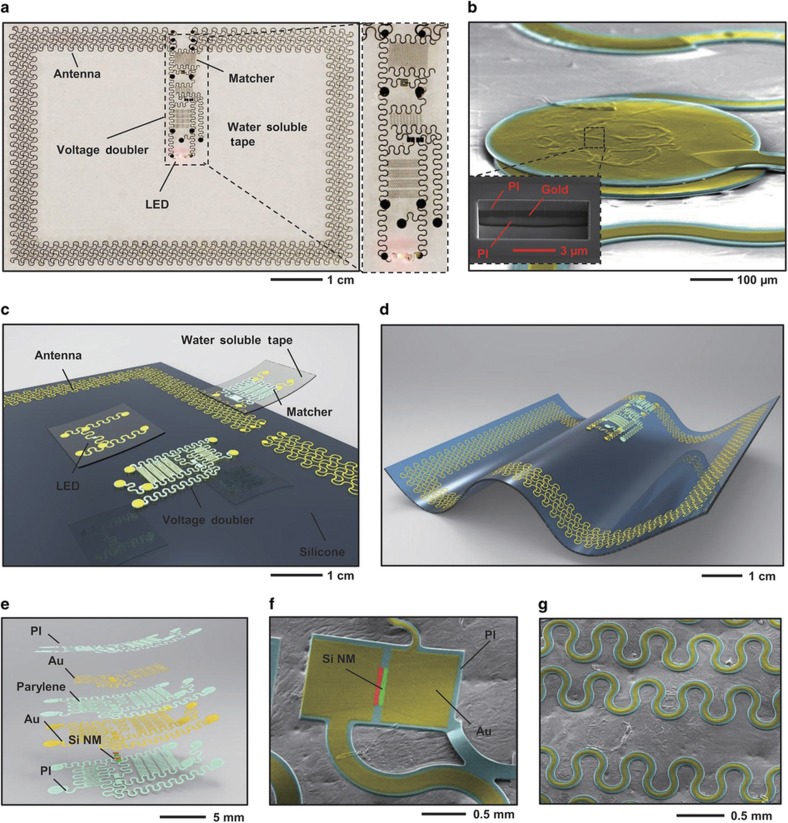
Schematic illustration and implementation of a modularized epidermal RF system for wireless power transfer. (**a**) Image of device while operating an integrated LED via power delivered by a remote RF source (15 W, 1.5 m). The loop antenna, formed with serpentine conductive traces in a square layout, spans the perimeter. The inset on the right highlights the collection of active components. (**b**) Top view SEM image of aligned gold pads whose electrical contact joins separate, laminated components. The inset provides a cross-sectional view of the interface. (**c**) Diagram that illustrates the modularization approach to device assembly, where sequential lamination of separately fabricated thin film components yields an integrated, functional system. (**d**) Diagram of a completed system on a thin silicone substrate. (**e**) Exploded view illustration of a voltage doubler. (**f**) Colorized SEM image of a silicon nanomembrane (SiNM) RF diode, integrated as part of a voltage doubler resting on a skin replica. (**g**) Colored SEM image of parallel plate capacitors in serpentine geometries on a skin replica.

Standard, monolithic strategies in design and fabrication limit the ability to optimize performance or conduct parametric studies of the analog RF circuits introduced here, where variability that arises from the uncertain dielectric properties of the surrounding biological tissues can cause significant complications. This circumstance demands a modularized approach to assembly, in which component fabrication and characterization occur before collective integration via soft-contact lamination processes (described in Supplementary Note 1) to yield functional systems for performance evaluation. This lamination can be conducted in a reversible mode for test/evaluation or in an irreversible mode for final device assembly. The latter relies on cold welding that occurs upon contact between exposed gold electrode pads on opposing surfaces^38^. One reversible variant involves the formation of a blocking self-assembly monolayer on the gold to prevent cold welding. In addition to control over the interface chemistries, we find that the kinetics of cold welding differ depending on the mechanical properties and geometries of the substrates. In all cases, lamination occurs without external pressure in a soft mode, in which the ‘wetting’ properties associated with low modulus elastomeric substrates^39^, and van der Waals forces^39,40^ drive contact between multiple, thin, soft electronic components to establish contacts. Although previously demonstrated in the context of organic electronic devices^38,39^, this same strategy works well with inorganic stretchable systems, as illustrated in studies of basic test structures (Supplementary Figure S1a). Here gold–gold cold welding that follows from contact at exposed pads yields low electrical resistance and robust operation under stretching. Observation of the gold–gold interface (Figure 1b) reveals homogeneous welding and intimate electrical contact.

### Modularized design

Figure 1a shows a modularized version of a completed device that includes an impedance matcher (Supplementary Figure S2a) connected to a loop antenna (Supplementary Figure S2b) and a voltage doubler (Supplementary Figure S2c), combined together via the soft-contact lamination technique (Figure 1b) described above. The load, for purposes of functional demonstration, is a small-scale LED (Supplementary Figure S2d). Each component incorporates stacked ultrathin layers of metals, polymers, and semiconducting materials (Figure 1e) in open-mesh serpentine layouts to yield soft, elastic mechanical properties for skin-mounted applications, using design principles described elsewhere^41^. Specifically, these components have total thicknesses between 6 and 10 μm, they exhibit effective linear elastic moduli of ~60 kPa for strains up to 6%, and they have area mass densities of ~10 mg cm^−2^ and thermal masses of ~7.6 mJ cm^−2^ K^−1^, all of which are comparable to the corresponding properties of the epidermis itself^41,42^. Modularization enhances the system-level yields and offers the ability to explore various combinations of components with different electrical properties and functions.

The overall device occupies an area of 5.1×4 cm^2^, dominated by the dimensions of the antenna. The inner area of the loop (4.2×3.1 cm^2^) defines the space for the other components. The antenna dimensions determine the overall sizes of these harvesting systems. Increasing the operating frequencies allows for size reductions. The antenna consists of circular serpentine metallic mesh structures (Supplementary Figure S2e) with inner radii of 340 μm and outer radii of 440 μm. The impedance matcher is an LC resonator. The inductor (Supplementary Figure S2f) consists of a three-turn square coil with traces that have widths of 70 μm, inner lengths of 130 μm and outer lengths of 570 μm. The capacitor uses a parallel plate design with a serpentine layout, in which Parylene-C (1 μm) serves as the dielectric (Supplementary Figure S2g). The length of each serpentine electrode is ~1.8 mm. The total number of electrodes lies between 2 and 16, thereby providing different capacitances. The impedance matcher (Supplementary Figure S2a) forms a low-pass filter circuit with the first capacitor in a voltage doubler (Supplementary Figure S2c), designed to match the impedance between the voltage doubler and antenna. The voltage doubler contains two p-intrinsic-n (PIN) diodes and two capacitors that each use 10 electrodes with an average length of ~0.8 mm, with Parylene-C (1 μm) as the dielectric. The wedge-shaped PIN diodes (Supplementary Figure S2h) each cover an area of ~0.2 mm^2^ and use single-crystalline nanomembranes of silicon (SiNMs) as the semiconductor. The half-wave rectification provided by the doubler converts an AC voltage output at the impedance matcher into a DC voltage with twice the magnitude to power the load circuit. Specifically, the PIN diodes function as switches to allow voltage to accumulate on C_1_ during only the negative cycle of the input voltage. Voltage doubling on C_2_ results from the use of C_1_ as an additional voltage source during the positive cycle of the input voltage. Moreover, C_2_ reduces fluctuations in the output voltage. The small-scale red LED that serves as the load is a de-packaged surface-mounted device (Rohm, Kyoto, Japan, PICOLED SML-P11).

Modularized assembly begins with transfer printing of the voltage doubler, registered to alignment marks on a membrane of polydimethylsiloxane (PDMS, Dow Corning, Midland, MI, USA, Sylgard 184; 20 μm in thickness) that supports the loop antenna. The impedance matcher then laminates between the antenna and the doubler. Attachment of the load circuit on the other end of the voltage doubler completes the assembly (Figure 1c). Slight variations in the impedance match between the loop antenna and the voltage doubler demand pre-integration tests that involve temporarily attaching impedance matchers with various capacitance values on a separate silicone substrate as an evaluation platform (Ecoflex, Smooth-on (Macungie, Pennsylvania, United State), thickness: 500 μm). Reversible lamination enables characterization of the power transfer efficiency with various combinations of matchers in different dielectric environments (for example, in air or on skin). The matcher that yields maximum output from the LED is irreversibly laminated without a silicone substrate together with a load circuit containing the LED, to the existing loop antenna and voltage doubler on the PDMS membrane (Figure 1d). Advanced mechanical design considerations^17^ yield characteristics that, as supported by observation under a scanning electron microscope (SEM) using a skin replica (Dragon skin, Smooth-on, Macungie, Pennsylvania, United State), enable the PIN diodes and capacitor traces to follow the topography of the skin (Figures 1f and g), indicating a high level of compliance and intimate skin contact.

### Fabrication of RF power transfer systems

The major processes involve fabricating separate functional components on a carrier wafer, releasing them for purposes of transfer printing temporarily to water soluble tapes, then to PDMS membranes, such that they can be integrated into modularized forms to complete the final systems. Fabrication of the voltage doubler starts with a silicon on insulator wafer (SOI; SOITEC, Bernin, France) that is thermally oxidized to form a doping barrier layer (~300 nm) (Supplementary Figure S13). Photolithography and reaction ion etching (RIE; Plasma-Therm, St Petersburg, FL, USA) defines a hard mask in the silicon dioxide in the geometry of the regions for doping. P-type doping occurs in a diffusion furnace (Intel, Santa Clara, CA, USA) at 1100 °C, with solid-state boron nitride as a dopant source (PDS, Saint-Gobain, Courbevoie, France). N-type doping with phosphorus involves the same procedures. Immersion in hydrofluoric acid (HF, 49%, Transene Company, Danvers, MA, USA) removes the silicon dioxide masking layer. RIE (CS 1701, Nordson, Westlake, OH, USA) creates an array of holes (3 μm diameter; 50 μm spacing) in the device silicon to facilitate the undercut of the buried oxide via buffered oxide etching in HF to form released silicon nanomembranes (SiNMs) on the carrier wafer. Transfer printing with a PDMS (Sylgard 184, Dow Corning, Midland, MI, USA) stamp delivers the SiNMs onto a partially cured polyimide layer (PI; PI2545, HD Microsystems, Parlin, NJ, USA; 110 °C for 1 min) spin cast on a wafer with an underlying, sacrificial coating of poly(methyl methacrylate) (PMMA 495, Microchem, Westborough, MA, USA; 60 nm). Further curing of the PI film for 1 min at 110 °C separates the PDMS stamp from the SiNMs. Removal of the PDMS stamp and full curing of the PI film (250 °C for 1 h in N_2_) with the SiNMs complete the transfer printing process. Photolithography and RIE then define wedge shapes in the SiNM for the RF diodes. Metal bilayers (25/300 nm of chromium/gold) deposited via electron beam evaporation form electrical interconnects in geometries defined by photolithography. A layer of parylene forms a dielectric for the parallel plate capacitor with the same metallization. RIE defines via holes through the parylene at designed locations to allow a connection between the top and bottom layers of metal (Cr/Au, 25/500 nm in thickness) for the capacitor plates, defined by photolithography and wet chemical etching. An additional layer of polyimide encapsulates the voltage doubler; photolithography and RIE define the final device layout. Immersion in acetone at 100 °C removes the PMMA to allow temporary retrieval of the devices onto water soluble tape (Aquasol Inc., North Tonawanda, New York, USA) and then onto a silicone substrate. The fabrication details of other components appear in Supplementary Note 6; the characterization details appear in Supplementary Note 7.

## Results and discussion

### Component performance

Measurements of the voltage doubler in Supplementary Figure S3a involve placement of the contact pads against a pair of probes connected to an RF signal generator, while another pair of probes connects the output near capacitor C_2_ to a high-speed oscilloscope (Supplementary Figure S3b). This setup determines the open-circuit voltage, with variable frequency input at powers of ~100 mW. As an example, three voltage doublers fabricated in a single batch yield voltage levels ranging from 0.8 V at 500 MHz to 0.3 V at 1 GHz (Supplementary Figure S3c). The modularized approach allows for the selection of devices with performance finely matched to optimize the overall performance.

**Figure 2:**
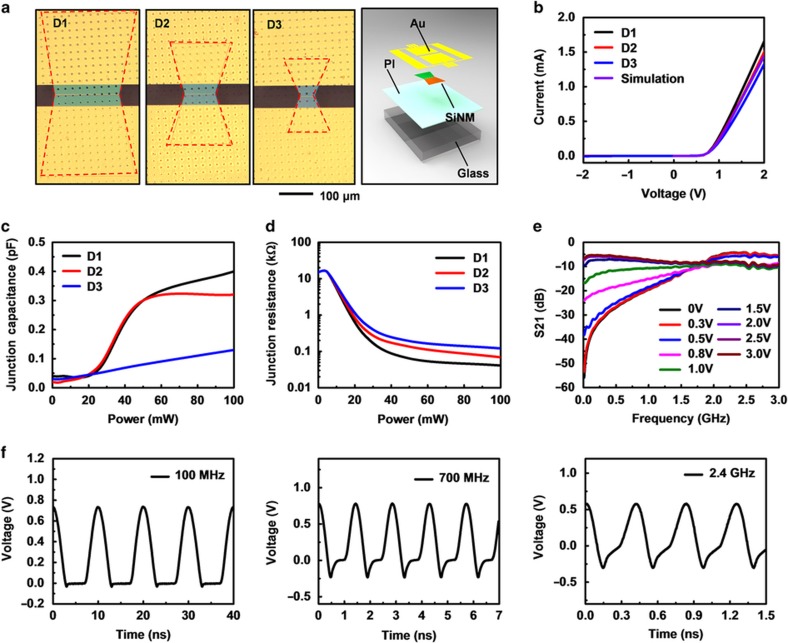
Electrical characterization of SiNM PIN diodes. (**a**) Optical images of PIN diodes that use SiNMs in wedge shapes with different geometries (D1, D2, D3). The frame on the left provides an exploded view of the schematic illustration. (**b**) Experimentally measured and simulated current–voltage curves associated with the diodes. The (**c**) junction capacitance, (**d**) junction resistance and (**e**) S21 parameters of the diodes. (**f**) Rectified voltages from the diodes at frequencies of 100 MHz (left), 700 MHz (middle) and 2.4 GHz (right).

The PIN diodes in the doublers incorporate single-crystalline SiNMs, with switching speeds and current densities that meet the requirements in high-frequency power applications. Wedge-shaped diodes (Figure 2a) with similar intrinsic region lengths (7 μm) but decreasing surface areas of 0.40, 0.15, and 0.17 mm^2^ for Diode 1 (D1), Diode 2 (D2), and Diode 3 (D3), respectively, and characterized through contacts to metal pads (Cr/Au, 10/300 nm thickness) in a ground-signal-ground, reveal current–voltage (*I*–*V*) responses that match those from simulation results (Silvaco, Santa Clara, CA, USA). The threshold voltages are 0.7 V, independent of design; the current levels (1.8, 1.6, and 1.4 mA at 2.0 V for D1, D2, and D3, respectively) scale with the surface areas of the diodes (Figure 2b). The junction capacitances are 0.40, 0.32, and 0.13 pF for D1, D2, and D3, respectively, at 100 mW input power, extracted using equivalent circuit models^43^ (Figure 2c and Supplementary Note 3), and are proportional to the surface areas. The junction resistances vary from 40.9, 69.0 to 120.8 Ω and are inversely proportional to the area (Figure 2d). D2 offers a favorable balance between junction capacitance and resistance. Rectification of RF signals at 100 MHz, 700 MHz, and 2.4 GHz in a half-wave circuit design yields voltage magnitudes of ~0.7, ~0.8, and ~0.5 V, respectively (Figure 2f). The frequency dependence follows from variations in the impedance of the diode and the matching between the diode and the test setup (Supplementary Figure S3d). Measuring the scattering parameters associated with RF signals that have a DC bias at the input and then decoupling this DC component at the output yields the switching speeds (Supplementary Figure S3e). The diode shows switching capability from conducting to non-conducting states up to 2.0 GHz, indicated by an increased S21 parameter with bias voltage levels larger than 0.5 V and a crossing point at 2.0 GHz (Figure 2e). Higher current levels in forward bias are possible with diodes that have rectangular shapes (Supplementary Figures S4a and b), but increased resistance associated with the correspondingly larger intrinsic areas can lead to high temperatures during operation (Supplementary Figure S4c).

**Figure 3:**
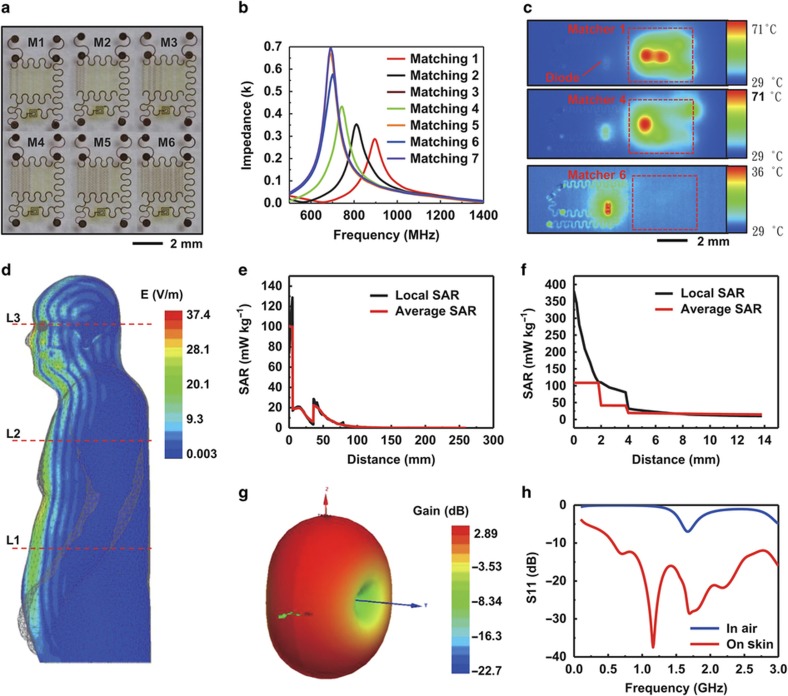
RF properties of modularized system components and results of SAR simulations. (**a**) Images of matching components with different capacitors, labeled from 1 to 6. (**b**) Resonance frequencies of these matching components. (**c**) Thermal analysis of the performance of matching components (1, 4 and 6; red dashed boxes) during RF operation in a voltage doubler. Low coupling efficiency manifests as high temperature during operation. (**d**) Simulated SAR across a model human body for the case of an RF source (Gain: 11 dBi, 15 W, 1 GHz) 1.5 m away from the human body. (**e**) Simulated SAR in the human body at L2. Average SAR is an average of local SAR over 1 g of tissue. (**f**) Simulated SAR in the skin area underneath an epidermal RF power transfer system, with a loop antenna mounted at L2. (**g**) Simulated radiation pattern of a loop antenna in air. (**h**) Simulated S11 parameter of a loop antenna evaluated in air and on skin. SAR, specific absorption rate.

Maximum conversion efficiencies of power received by the antenna to DC output by the voltage doubler can be realized via comparative analysis of the performance achieved with impedance matchers (labeled from M1 to M6) that offer different capacitance values to compensate for different dielectric environments (Figure 3a). The different capacitances, achieved by changing the numbers of serpentine parallel-plate capacitors, lead to resonant frequencies between 1 GHz and 700 MHz (Figure 3b). Operation while performing thermal infrared (IR) imaging can reveal heating and associated losses in the matchers. As shown in Figure 3c, temperature increases (Δ*T*~42 °C and 34 °C) occur at the inductors on M1 and M4, with little heating in the diodes (~30 °C), consistent with ineffective coupling. Comparatively small temperature changes occur in M6, indicating high energy conversion efficiency.

### Simulations of the specific absorption rate

Finite element modeling (Ansys HFSS) can quantify the specific absorption rate (SAR) in the human body during exposure under conditions similar to those used in RF power transfer experiments. For a transmission antenna operating at 15 W (Figure 3d), the electrical field distribution within a human body model was obtained at a distance (1.0 m) near the boundary of an electromagnetic wave, which is considered as the strongest electric field in far-field applications. The magnitude of the field reaches a maximum near the eyes, which corresponds to the largest SAR value (~410 mW kg^−1^). Three body positions, indicated by L1 (equal to the level of the antenna), L2, and L3, further illustrate the spatial distribution of the SAR (Figure 3d). The local SAR was ~124 mW kg^−1^ and 17.2 μW kg^−1^ in the front and back part of the model, respectively, at L1 (Figure 3e). The SAR in subcutaneous tissues, such as fat and muscle, yields different levels of attenuation, determined by the dielectric properties of these tissue layers. The simulated SAR in all cases (Supplementary Figures S5a and b) is much lower than the limits associated with FCC guidelines (1.6 W kg^−1^)^44^. The skin directly underneath the device has some additional SAR from the loop antenna. Calculation from the Friis transmission equation shows a theoretical, ideal RF power available on the skin region at a distance of 1.5 m away from the transmission antenna (Supplementary Note 4). This power is largely absorbed by the loop antenna on the skin, whereas only a small portion is reflected. Similarly, simulations of the electrical field distribution underneath the loop antenna (Supplementary Figure S5c) reveal the largest field magnitudes near the locations of the antenna patterns. According to this simulation, the local SAR value associated with reflections from the loop antenna is 380 mW kg^−1^ (Figure 3f), which is also smaller than the partial body FCC limit.

### Antenna design and characterization

The epidermal loop antenna has an expected donut-shaped radiation pattern with maximum and minimum gain values in the plane and out of the plane of the antenna, respectively (Figure 3g), as determined by simulation or Supplementary Equations S1.1 to S1.3 (Supplementary Note 2). The gain is ~2.89 dB in air (Supplementary Figure S6a) at a frequency of 1.65 GHz, corresponding to the dip in the S11 curve (Figure 3h). This gain significantly decreases (approximately −18.5 dB) upon placement on the skin (Supplementary Figures S6b and c), consistent with simulations that show that the S11 parameter significantly changes owing to the dielectric properties of the skin (Figure 3h). The strong dependence of key properties on the surrounding dielectric characteristics emphasizes the importance of modularization schemes for efficient impedance matching.

Measurements of the antenna under uniaxial and biaxial strain define variations in properties with physical deformation. In particular, a network analyzer can capture the magnitude and phase of the S11 parameters of the antennas under different strain levels up to 20%. The S11 parameters can be used to calculate the impedance of the antenna using the equations in Supplementary Note 5.

For a loop antenna with dimensions of 5.1×4 cm^2^, the fundamental resonance frequency in the magnitude of the S11 parameters decreases consistently from ~1.01 to 0.96 GHz for strains along *x* (5.1 cm) and in a biaxial mode (Supplementary Figures S7a and c). Only small changes (<6 MHz) occur for the same levels of strain along *y* (4 cm) (Supplementary Figure S7b). The corresponding calculated impedances at these resonance frequencies vary from only 19–13 Ω (Supplementary Figure S7d), thereby illustrating stable performance under mild stretching.

The small difference in the dependence of the impedance on stretching along *x* and *y* may be due to the elongated overall geometries, that is, lengths of 5.1 and 4 cm in these two directions (Supplementary Figures S7d–f). Shifts in the fundamental resonance frequencies (~1.0 GHz) are smaller than those in the harmonic modes (~1.6 GHz). In addition, stretching in one direction is accompanied by compression in the other direction, resulting in small changes in area and circumference. Thus, the values of *R*_r_, *R*_L_ and *L* undergo minimal changes according to Supplementary Equations S4.4 to S4.7 (Supplementary Note 5), in which area and circumference have major roles in determining the impedance and resonance frequency of the antenna.

The response of a meander dipole antenna with strain provides a useful point of comparison. The anisotropic design of the dipole antenna suggests a distinct response of the S11 parameters and the impedance, with stretching along the poles. As shown in Supplementary Figure S8, small frequency shifts in S11 parameters occur for strain along *x* (5.3 cm), whereas larger shifts accompany strains along *y* (2.0 cm) and in biaxial modes. As variation in the effective length of the antenna in the *x* direction is much smaller than in the effective length along the *y* direction, the antenna properties show larger changes during the *y* direction stretching.

The Q factor of the antennas is ~5.1. The impedance of the loop antenna can be first determined by considering an equivalent model that contains serially connected radiation resistance, loss resistance, and inductance. These components, as well as the overall impedance, can be determined using Supplementary Equations S4.1 to S4.3 (Supplementary Note 5).

### Mechanical properties

**Figure 4:**
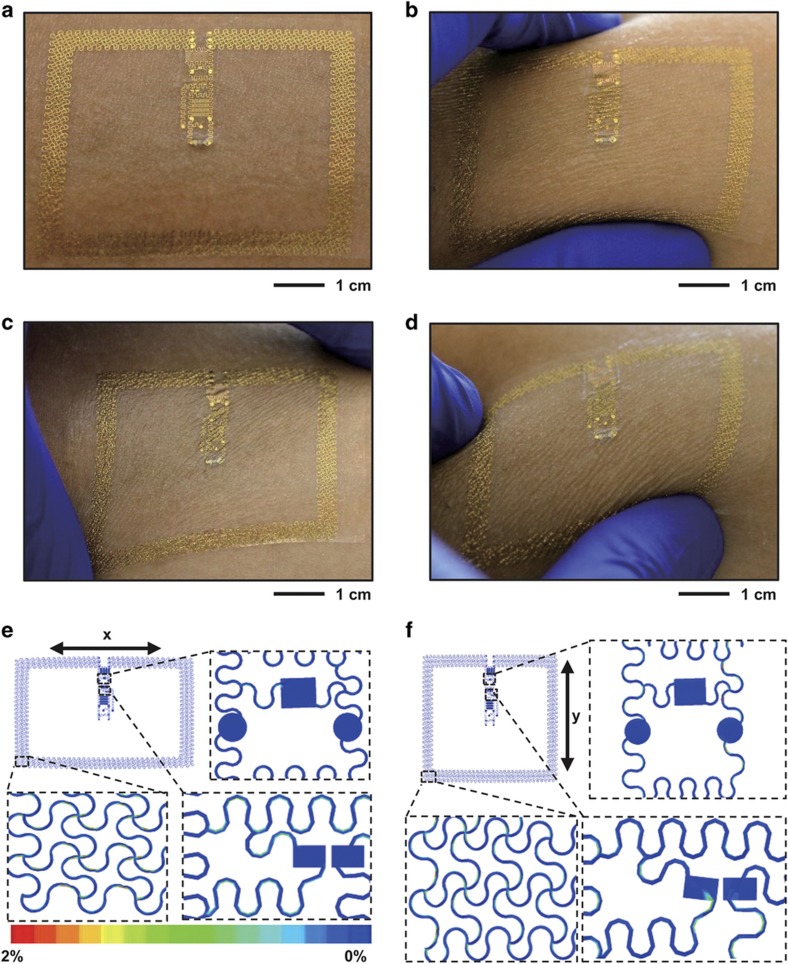
Mechanics of an epidermal RF system. Pictures of an epidermal RF system integrated on the skin (**a**) in its native state, (**b**) during compression by pinching (**c**) under uniaxial stretch and (**d**) while twisted. Finite element simulation of the distributions of strain under 20% uniaxial stretching in the (**e**) *x* and (**f**) *y* directions.

The fully integrated systems have high levels of flexibility and stretchability owing to their overall layouts (Figure 4a)^16,17^, with an ability to accommodate extreme deformations of the skin, including uniaxial stretching (Figure 4b), biaxial (Figure 4c) stretching and squeezing (Figure 4d). Mechanical simulations using finite element analysis techniques (ABAQUS)^45^ indicate that for 20% uniaxial stretching in the *x* and *y* directions, the strains in the electronic materials remain <2% (Figures 4e and f), with the largest strains at the corners of the loop antenna. The calculated strains are lower than the fracture strain of copper (~5%), revealing that the total biaxial stretchability of the epidermal RF system is larger than 20%. Assuming a yield strain of ~0.3% in copper, the elastic stretchability in both directions is ~6% (Supplementary Figures S9a and b).

### Operation in RF power transfer

**Figure 5:**
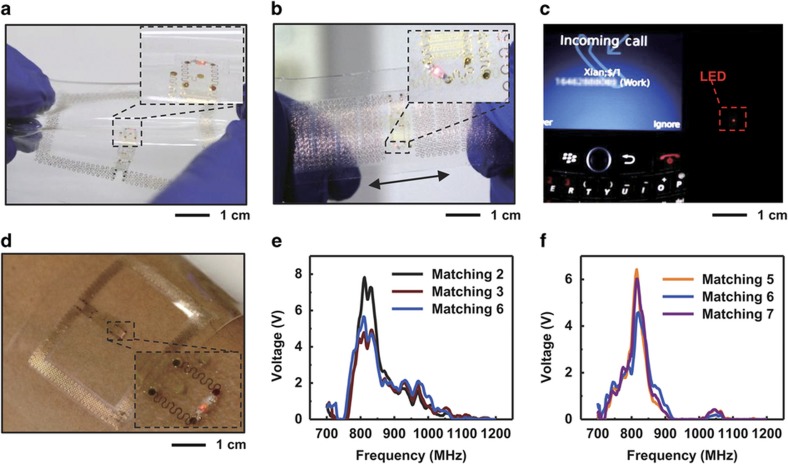
Demonstration of RF wireless power transfer. Epidermal RF system operating while (**a**) twisted and (**b**) repeatedly stretched. (**c**) Demonstration of the use of an epidermal RF system to capture RF output from a cell phone to supply power to an LED. (**d**) Epidermal RF system powering a red LED while on the skin using RF transmitted by a remote source (15 W, 1.5 m, 700 MHz–1.5 GHz). Open-circuit voltage output (**e**) in air and (**f**) on skin when implemented with different matching components.

The systems operate effectively even during deformation, with stable power output under significant twisting and stretching (Figure 5a) for both the loop and meander dipole antennas (Supplementary Figure S10). Sufficient RF power can be captured for the operation of an integrated LED in both static (Supplementary Figure S11) and stretched states (20% strain, repeated; Figure 5b and Supplementary Movie S1) for the case of a transmission antenna (700–2500 MHz; 11 dBi) emitting 15 W of RF power (~1.0 GHz) at distances of up to 1.5 m. A device mounted on a phantom skin substrate^46^ and illuminated with similar RF power reveals only a small increase in temperature (~0.4 °C; IR camera, Supplementary Figures S12a and b). Together with the SAR simulation results described above, this finding supports safe operation under FCC regulations. Under these conditions, the open-circuit voltage of the RF system is ~8 V in air (Figure 5e) and ~6.5 V on skin (Figure 5f), which is sufficient for operating circuits with simple sensing and communication functions^47^. An RF power transfer system integrated with a loop antenna can power an LED on the skin in continuous and pulsed modes (Figure 5d and Supplementary Movie S2). Devices can also be designed to allow similar operation based on the RF output of a cell phone antenna (AT&T, 850 MHz; Blackberry, 8520; Figure 5c and Supplementary Movie S3).

## Conclusion

The results reported here establish the materials, designs, and integration strategies for an epidermal wireless RF power transfer system. This option for battery-free operation complements other approaches, adding flexibility in system-level design. Within exposure guidelines, sufficient power can be received for operating the many different components needed in epidermal technologies, such as radios, sensors, memory devices and low power microprocessors. Further improvements in the harvesting capabilities will follow from advances in antenna designs to increase angular bandwidth and to reduce losses associated with RF coupling to the skin. The addition of a modest amount of storage, either in the form of supercapacitors or chip-scale batteries, will help to eliminate effects of intermittency in the received power. The present work establishes a baseline for on-going efforts to develop practical applications. The power transfer system in its present format can be exploited in applications such as optogenetics, oximetry, and phototherapy.
