# Supplementary material for: Epidermal radio frequency electronics for wireless power transfer
Source: Microsyst Nanoeng. 2016 Oct 24;2:16052. doi: 10.1038/micronano.2016.52 (PMC6444737; doi:10.1038/micronano.2016.52)
Supplement: Supplementary Information [file micronano201652-s1.pdf]

Supplementary file

Epidermal radio frequency electronics for wireless power transfer

Xian Huang[1,*], Yuhao Liu[2,*], Gil Woo Kong[2], Jung Hun Seo[3], Yinji Ma[4,5,6], Kyung-In Jang[2,7], Jonathan A. Fan[7,8], Shimin Mao[2], Qiwen Chen[2], Daizhen Li[2], Hank Liu[2], Chuxuan Wang[2], Dwipayan Patnaik[2], Limei Tian[2], Giovanni A. Salvatore[2], Xue Feng[5], Zhenqiang Ma[3], Yonggang Huang[4,6] and John A. Rogers[2]

Microsystems & Nanoengineering (2016) **2,** 16052; doi:10.1038/micronano.2016.52; Published online: 24 October 2016

SUPPLEMENTARY NOTE 1: SOFT-CONTACT LAMINATION

Methods in soft lamination are known to provide efficient electrical contacts in organic transistors[1,2], solar cells[3], and carbon nanotube-based passive integrated devices[4]. Instead of conventional adhesives[5], mechanical compression bonding techniques[6], thermal annealing approaches[7], or surface chemistry modifications[8], soft-contact lamination exploits the 'wetting' properties that are obtained from low modulus elastomeric substrates, van der Waals forces[1,9] and cold welding of thin gold films[10]. The results establish conformal, microscopic-scale electrical contacts in ambient environment.

Reversible Approach

This approach exploits only the 'wetting' properties from low modulus elastomeric substrate and via van der Waals forces to provide temporary low resistance electrical contact between two metal pads transfer-printed on a silicone substrate. We use this method mainly for impedance matching tests between antennas and doublers, in which the matchers are transfer-printed on separate silicone substrates. The process begins with transfer-printing of an electronic component on a thin silicone substrate (PDMS, 20 μm), then delivering an electronic component on another comparatively thicker silicon substrate (PDMS, 500 μm). Treatment of the latter with ethanoethiol (Sigma Aldrich, St Louis, MO US) vapor for 5 to 10 s creates a self-assembled monolayer of alkyl thiolates at the gold-gold interface to prevent cold welding[10]. Alignment and lamination of the metal connector areas in two circuit components spontaneously bring them into physical and electrical contact without external applied pressure and heat treatment. The roughness, cleanliness and surface chemistry of silicone substrate may affect electrical contact results. A large surface area of silicone substrate improves 'wetting' and spontaneously attachment.

Irreversible Approach

The permanent construction of wireless RF power system by irreversible soft-contact lamination begins with the fabrication of individual circuit components on wafer substrates, and ends with integration of these components via an intrinsically irreversible cold welding process. The assembly starts with transfer-printing an electronic component on a silicone substrate, similar to the reversible approach. Then, an electronic component is delivered onto a temporary water soluble tape (Aquasol, cellulose) without a silicone substrate, and contacts with the existing component on the silicone substrate. Alignment of the circular metal pads in two circuit components brings them physical and electrical contacts under ambient air pressure, room temperature, and the presence of volatile organic contaminants. With small applied contact pressure (<10 to $20\,\mathrm{N\,m^{-2}}$)[10,11], layers of nanoscale metallic atoms based on same[12–14] or different[15,16] materials can be bonded together. In a test structure (Figure S1a), a 20 nm gold ultrathin film deposited on bonding pad surfaces formed the atomic layers; the underlying compliant elastomeric supports facilitate the atomic contact at gold-gold interface between bonding pads. This process allows fast surface diffusion of metal atoms, forming strong adhesion between two nanoscale metal layers in close contact.

SUPPLEMENTARY NOTE 2: ANTENNA DESIGN

Loop antennas, which are used extensively in high frequency and ultra-high frequency bands, meet the key requirements for operation in the epidermal RF power transfer system. Meander dipole antennas are also of interest, due to the ease of adjusting their operating frequency by changing their lengths. Both types of antennas are considered electrically small, where the overall length is less than one tenth of the wavelength of the RF radiation at their resonant frequencies. Although these antennas have low radiation efficiency, they are suitable as receiver antennas in the systems described here. Experimental characterization can be accomplished through measurements with a network analyzer. Finite element analysis software (HFSS) can be used to further explore the properties, including the radiation patterns.

The rectangular loop antenna in Figure S1b has an effective length of a (3.5 cm) and an effective width of b (4.6 cm). Assume that a current $I=I_0e^{-i\omega t}$ flows uniformly around the loop, where ω is the angular frequency. For an observer at angles (θ, ϕ) with respect to the z axis (in a spherical coordinate system with origin at the antenna), the unit wave vector has rectangular components.

$$\hat{k} = \sin\theta\cos\phi\hat{x} + \sin\theta\sin\phi\hat{y} + \cos\theta\hat{z} \qquad (S1.1)$$

For a small loop, where a and b are much smaller than λ, the wavelength of the RF radiation, the electric field of the loop

[1]Department of Biomedical Engineering, School of Precision Instrument and Opto-electronics Engineering, Tianjin University, Tianjin 300072, China; [2]Department of Materials Science and Engineering, University of Illinois at Urbana-Champaign, Urbana, IL 61801, USA; [3]Department of Electrical and Computer Engineering, University of Wisconsin-Madison, Madison, WI 53706, USA; [4]Department of Civil and Environmental Engineering, Northwestern University, Evanston, IL 60208, USA; [5]Department of Engineering Mechanics, Center for Mechanics and Materials, Tsinghua University, Beijing 100084, China; [6]Department of Mechanical Engineering, Northwestern University, Evanston, IL 60208, USA; [7]Department of Robotics Engineering, Daegu Gyeongbuk Institute of Science and Technology, Daegu 42988, Republic of Korea and [8]Department of Electrical Engineering, Stanford University, Stanford, CA 94305, USA.
Correspondence: John A. Rogers (jrogers@illinois.edu)
*These authors contributed equally to this work.

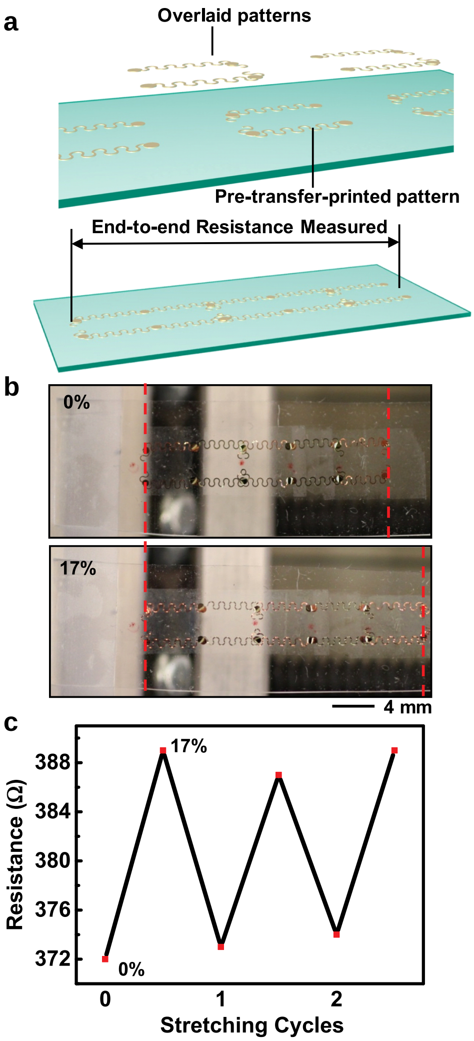

Figure S1 Demonstration of structure cold welding using parallel serpentine conductive traces with contact pads. (**a**) Schematic illustration of transfer-printed pairs of test structures on a PDMS substrate. (**b**) A completed test structure during uniaxial stretching to strains of 0 and 17%. (**c**) Resistance measured through repeated cycles across the test structure at 0 and 17% stretching.

antenna can be expressed as

$$E = \quad \hat{\phi} k^2 I_0 ab \frac{e^{i(kr \quad \omega t)}}{cr} \sin\,\theta \qquad (S1.2)$$

where c is the speed of light. For a rectangular loop, the radiation pattern is

$$\frac{dP}{d\Omega} = \frac{8 I_0^2 \sin^2(\frac{ka}{2}\sin\,\theta\cos\phi)\sin^2(\frac{kb}{2}\sin\,\theta\cos\phi)}{\pi c \sin^2\theta \sin^2 2\phi} \qquad (S1.3)$$

SUPPLEMENTARY NOTE 3: ELECTRICAL ANALYSIS OF SINM DIODES

The diode in the voltage doubler adopts a PIN diode format. The dielectric relaxation frequency of the diode can be determined from the following equation:

$$f_{r,\mathrm{GHz}} = 1/(2\pi\varepsilon_0\varepsilon_R\rho_{\Omega \quad cm}) \qquad (S2.1)$$

where ε_0 is the vacuum permittivity, ε_R is the relative permittivity of silicon and ρ is the resistivity of the silicon. The optimum operating frequency for the PIN diode is at least three times higher than the dielectric relaxation frequency. The performance of the PIN diode is determined by its i-region resistance R_I, junction resistance R_j, and junction capacitance C_j. The values of these parameters can be individually determined by the following equations.

$$R_I = 4V_T \sinh(\frac{W}{2L})[\tan^{\ 1}(\exp(\frac{W}{2L}) \quad \frac{\pi}{4})]/I_0 \approx \frac{W^2}{2\mu_{AP}\tau I_0} \qquad (S2.2)$$

$$R_J(f) = \frac{KT}{qI_0}\beta\tanh(\frac{W}{2L})\cos(\phi \quad \frac{\theta}{2}) \qquad (S2.3)$$

and

$$C_j = \frac{\varepsilon_0\varepsilon_R A}{W} \qquad (S2.4)$$

where A is the cross section area of the i-region and W is the i-region thickness. A PIN diode, designed for high frequency operation, is usually fabricated to have low capacitance, because the reactance of the diode in the OFF condition must be large compared to the line impedance. The ratio of the PIN's area to thickness is adjusted to obtain the desired capacitance. The resistivity or doping level of the i-layer is not critical as long as it is greater than 20–50 Ohm-cm for operation at 1 GHz. The transit time and the relaxation frequency requirements are easily obtained.

SUPPLEMENTARY NOTE 4: SAR SIMULATION

The power available at the loop antenna is calculated based on Friis transmission equation,

$$\frac{P_r}{P_t} = G_t G_r \left(\frac{\lambda}{4\pi L}\right)^2 \qquad (S3.1)$$

where P_t is the transmitted power, P_r the received power, G_t the transmit antenna gain, G_r the loop antenna gain, L is the distance between two antennas, and λ the wavelength of the RF radiation. For present experiments, P_t is 15 W, G_t is 11 dBi and G_r is −0.15 dBi for operation on skin as determined by simulation using HFSS. The power received by the loop antenna at a distance of 1.5 m from the transmit antenna is then determined to be 0.034 W at a frequency of 1.16 GHz. Only a small part of the received power leaks into the surrounding environment through the loop antenna, the percentage of this part of emitted energy is determined by the reflection coefficient (Γ) of the antenna, which is 0.235 according to the simulation result from HFSS. As a result, for 0.034 W input power, only 1.89 mW of power will be reflected.

SUPPLEMENTARY NOTE 5: ANTENNA SCATTERING PARAMETER AND IMPEDANCE CALCULATION

The S11 parameters can be used to calculate the impedance of the antenna using the following equations:

$$R + jX = |S11|\cos\theta + j|S11|\sin\theta \qquad (S4.1)$$

$$Z_{\mathrm{real}} = Z_0\left(\left(1 \quad R^2 \quad X^2\right)/\left(\left(1 \quad R\right)^2 + X^2\right)\right) \qquad (S4.2)$$

$$Z_{\mathrm{img}} = Z_0\left(2Xj/\left(\left(1 \quad R\right)^2 + X^2\right)\right) \qquad (S4.3)$$

in which, |S11| and θ are the magnitude and phase of the S11 parameters measured by the network analyzer. R and X are the real part and imaginary part of the S11 parameters, and Z_{real} and Z_{img} are the real part and imaginary part of the complex antenna impedance.

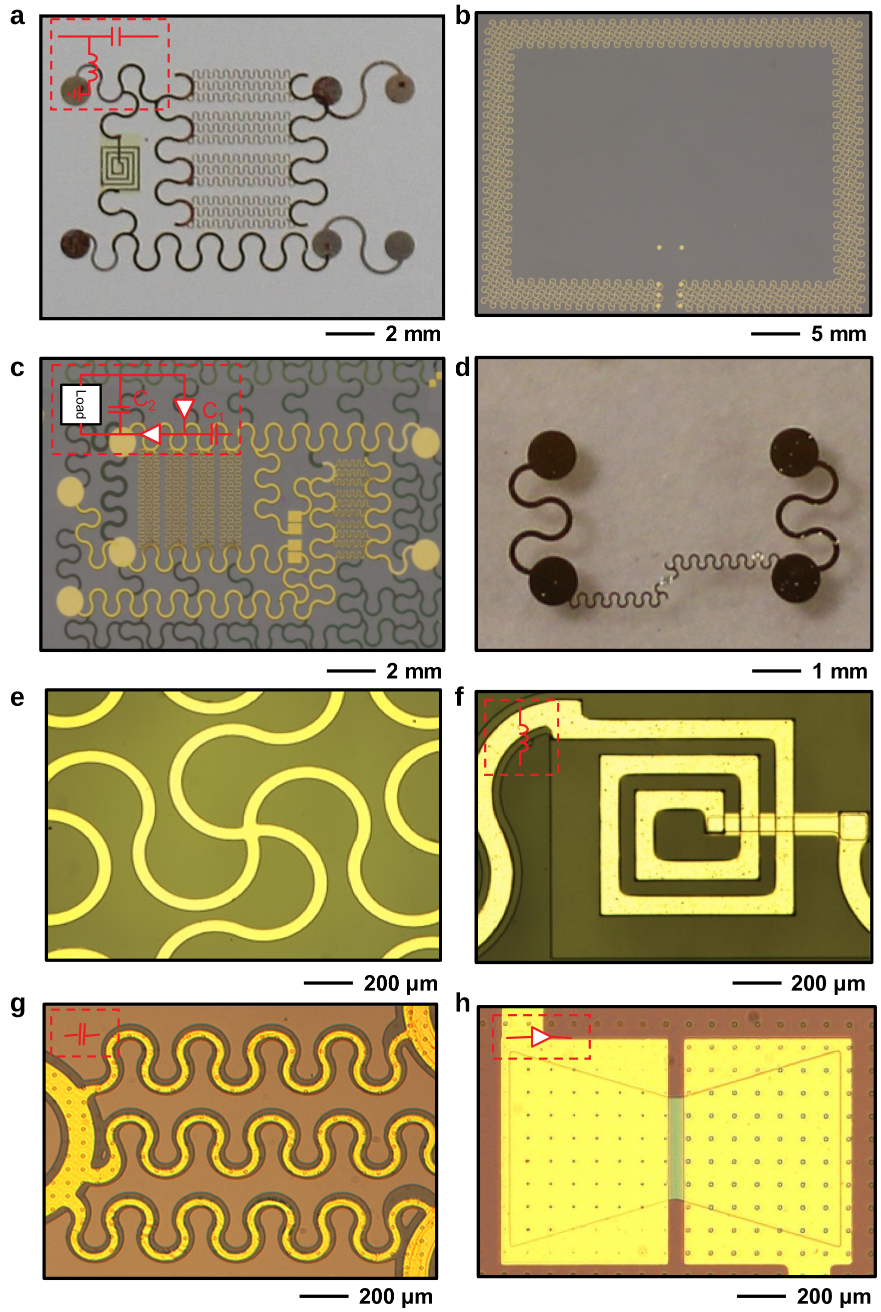

Figure S2 Individual components of an epidermal RF system, including (**a**) an impedance matcher, (**b**) a loop antenna, (**c**) a voltage doubler, and (**d**) a load circuit with a LED. Enlarged views of the above mentioned components include (**e**) mesh structures of the loop antenna, (**f**) an inductor in the impedance matcher, (**g**) capacitors in voltage doubler, and (**h**) A SiNM PIN diode.

These components as well as the overall impedance can be determined using the following equations:

$$Z_{in} = R_r + R_L + j\omega L \quad (S4.4)$$

$$R_r = 31200\left(S/\lambda^2\right)^2 \quad (S4.5)$$

$$R_L = \frac{2(l_1 + l_2)}{2\pi a}\sqrt{\frac{\omega\mu}{2\sigma}} \quad (S4.6)$$

$$L = \frac{\mu_0}{\pi}\left[l_2\cosh{}^{1}\frac{l_1}{2a} + l_1\cosh{}^{1}\frac{l_2}{2a}\right] \quad (S4.7)$$

where R_r and R_L are the radiation resistance and the loss resistance of the loop antenna, L is the inductance of the antenna. S is the area of the antenna, l_1 and l_2 are the lengths of the loop antenna in x and y direction, a is the wire radius, ω, μ, σ are angular frequency, permeability, and conductivity, respectively.

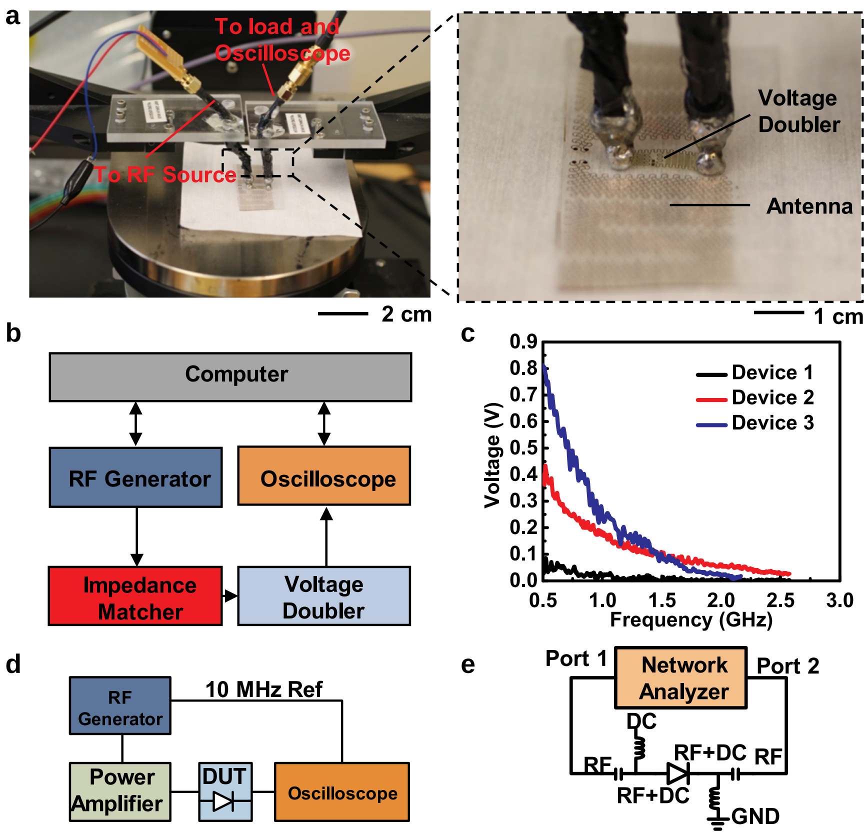

Figure S3 Characterization of an epidermal RF power system. (**a**) Setup for testing a voltage doubler on a probe station, in which two pairs of customized probes connect a source and a load to the voltage doubler. (**b**) A block diagram of the characterization system. (**c**) Measured voltage output from three randomly selected doublers. (**d**) A block diagram for testing the rectification of PIN diodes. (**e**) An equivalent circuit to test switching properties in PIN diodes using a network analyzer with DC bias and capacitors to decouple DC signals.

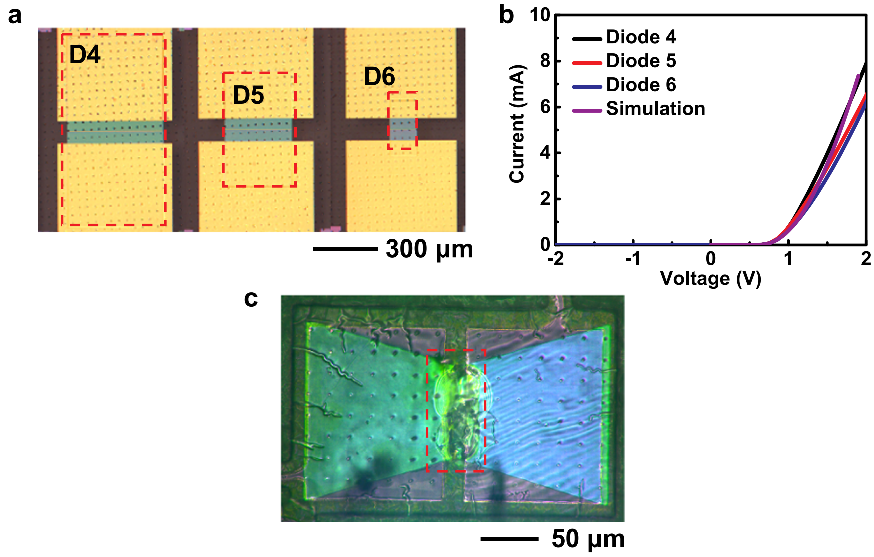

Figure S4 Characterization of PIN diodes based on silicon nanomembrane. (**a**) D4, D5 and D6 correspond to PIN diodes with widths of 500, 350 and 150 μm. (**b**) Measured current-voltage (IV) curves and simulated result using Silvaco. (**c**) Diode breakdown at high temperature due to excessive current density.

SUPPLEMENTARY NOTE 6: DEVICE FABRICATION

The RF power system is an integrated system composed of an antenna, impedance matcher, voltage doubler, connector and an LED (PicoLED, SML-P11, Rohm Semiconductor, Kyoto, Japan) (Figure S2). All modularized components involve different fabrication procedures, each starting with silicon wafers (University Wafers, 3″, 16–22 Ohm/cm) as bare substrates. Poylmethylmethacrylate (PMMA 495 A2, MicroChem, Westborough, MA USA) and polyimide (PI) (PI2545, HD Microsystems, Parlin, NJ USA) spin cast onto these wafers serve as sacrificial layers whose elimination releases the completed components for integration onto an silicone substrate.

Both the loop antenna and the load circuit are fabricated starting with a laminate (Microthin, Oak Mitsui Inc., Hoosick Falls, NY USA) that contains a double stack of copper foil (5 and 17.5 μm in thickness) separated by a releasing layer. Spin casting defines a film of polyimide (1 μm in thickness) on the side with the thin copper. The cured polyimide can be peeled, with the thin copper layer attached, from the thick copper layer and the releasing layer, for subsequent attachment onto a layer of PDMS spin-cast on a glass slide. The exposed thin copper layer is coated with a bilayer of Ti/Au (5 and 25 nm, respectively). Photolithography followed by metal etching defines the patterns of the loop antenna or the load circuit. Another spin-casting process defines a layer of PI on the resulting patterns. Photolithography and reactive ion etching (RIE) to define the overall patterns of the antenna and the load circuit. Removal of the photoresist mask completes the fabrication of these two structures. Retrieval onto the surface of a piece of water soluble tape allows for further assembly and integration.

The impedance matching circuit uses the same type of copper laminate and methods similar to those described above for the loop antenna and the load circuit to define the bottom electrodes of the parallel plate capacitors and the coil. A layer of parylene (1 μm) deposited on the circuit layer serves as a dielectric layer. RIE defines openings that serve as electrical vias to connect the copper layer with a deposited bilayer of Cr/Au (5 and 300 nm, respectively). The entire matching circuit is then covered with additional PI layer that is patterned to complete the device.

Assembly of the components to build working systems occurs on a thin (20 μm) layer of PDMS spin cast on a water soluble tape on a glass substrate. All components are coated with a bilayer of Ti/SiO_2 (5 and 40 nm, respectively). Exposure to UV induced ozone creates hydroxyl groups for permanent covalent -Si-O-O-Si- bonding at the contacting interfaces. Transfer printing of individual components onto the PDMS occurs in order, starting with the antenna, the voltage doubler, the impedance matching circuit, and the load. Rinsing the resulting device with water allows the removal of water soluble tapes on the device. The completed device is transferred to another water soluble tape on a glass slide, followed by removal of the first layer of water soluble tape. The system is then ready to be integrated on the skin.

SUPPLEMENTARY NOTE 7: EXPERIMENT METHODS

RF Harvesting and Characterization

Characterization of the RF power system is conducted using a signal generator (Agilent N5181A, 100 kHz–6 GHz), a power amplifier (Empower 1119-BBM3K5KHM, 500 MHz–2500 MHz), and a wideband antenna (Wilson 204475, 700 MHz–2700 MHz). The signal generator defines the RF frequency and the pulse waveform, while the amplifier boosts the signal by 48 dB to an output power of 15 W. The wideband antenna provides stable gain that varies only slightly around 11 dBi throughout the frequency band from 700 to 2500 MHz. Testing of the individual components occurs prior to assembly. For example, a network analyzer (Agilent E5062A, 300 kHz–3 GHz) yields the S parameters and impedance values of the loop antennas; an impedance analyzer (Agilent 4291A, 2 MHz–1.8 GHz) reveals properties of the matchers. Setup shown in Figure S3 uses two probes to inject 100 mW RF power into the voltage doubler from RF signal generator, and two additional probes connect with the output of the doubler to

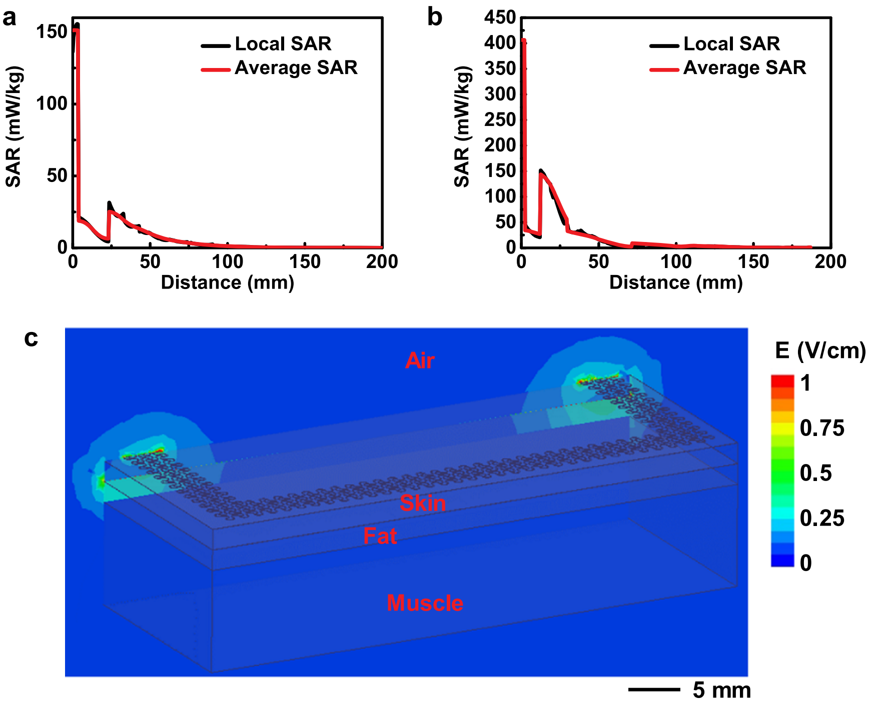

Figure S5 SAR simulation and analysis of a loop antenna. (**a**) Simulated local and average SAR results along L2 in Figure 3d. (**b**) Simulated local and average SAR results along L3 in Figure 3d. (**c**) SAR simulation of a loop antenna placed in contact with the skin.

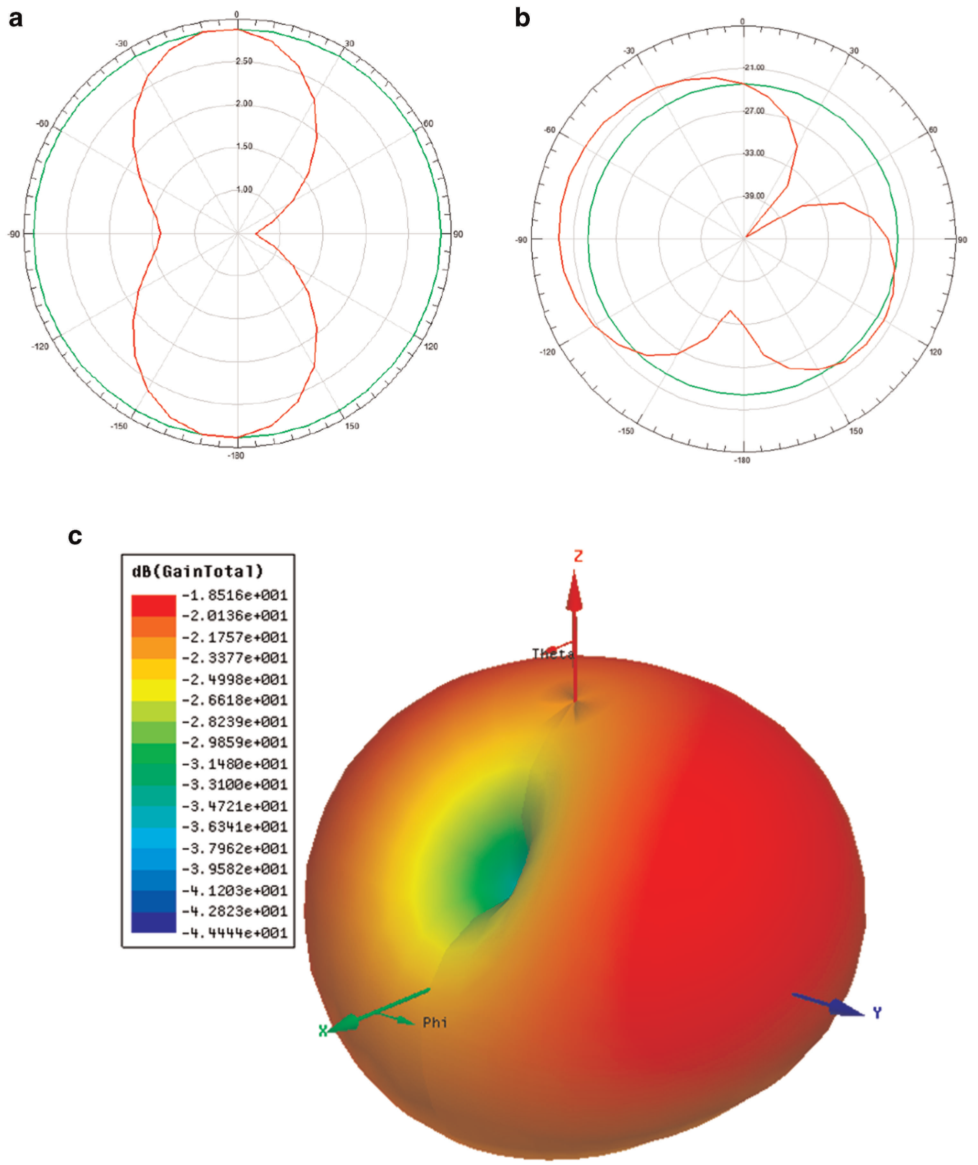

Figure S6 Simulation and analysis of the gain of a loop antenna (**a**) Loop antenna radiation pattern in air. (**b**) Loop antenna radiation pattern on skin. (**c**) Simulated Loop antenna gain on skin.

measure its open circuit voltage using an oscilloscope (Agilent DSO8104A, 1 GHz, 4 GSa s^{-1}).

S-parameter Measurement

Measurement of the S parameters of the diodes uses a network analyzer (Agilent E8364A 45 MHz–50 GHz), calibrated using a standard Short-Open-Load-Thru (SOLT) on-wafer probing kit with a frequency range of 45 MHz–50 GHz. The RF signal and DC bias between 0 to 3 V are applied at a fixed RF power of −5 dBm to determine the insertion loss (Figure S3). The measurements involve DC isolation characteristics of diodes. The diode transfer curves are simulated using Silvaco software.

PIN Diode Rectification Test

For measurement of the rectifying characteristics of the diodes, an Agilent 8648D Synthesized RF Signal Generator (9 kHz to 4 GHz) is used to supply signal combined with RF and DC components (Figure S3d). A RF power amplifier (SPA-120-18-003-SMA, 18 dB gain broadband amplifier operating in the 100 MHz to 12 GHz frequency range) boosts the output power to 100 mW. The output RF characteristics are obtained by a Wide-Bandwidth Oscilloscope (Agilent 86100A Infiniium DCA). The two systems share a 10 MHz reference signal to synchronize the phase. All systems are connected by RF cables (Gore cable, performed up to 50 GHz).

Mechanical Simulation

A commercial finite element analysis software (ABAQUS) allows computational study of the stretchability of the systems (Figure S9). The structures and materials used in four simulated components are combined to simplified models that facilitate simulation. The structures include 2 μm/1 μm of Cu/PI for the antenna, and 2 μm/1 μm/1 μm of PI/Au/PI for the voltage doubler, the matcher, and the load circuit. These layers are modeled by the composite shell element (S4R), while the silicone elastomer (PDMS, ~20 μm in thickness, with modulus ~60 kPa) and the skin (~1 mm in thickness, with modulus ~130 kPa[17]) is modeled by the hexahedron element (C3D8R)[18].

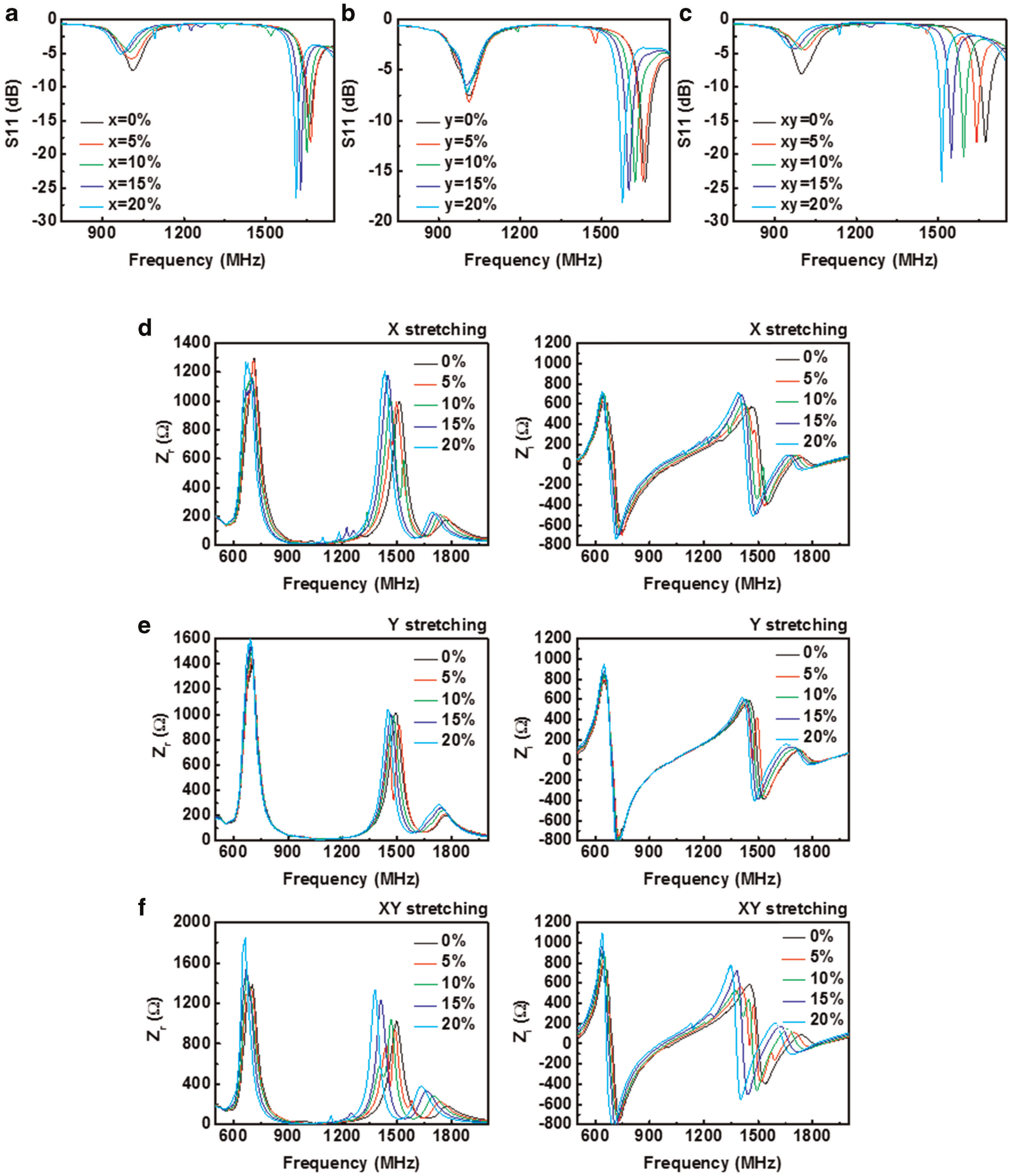

Figure S7 S-parameter measurement and impedance simulation of a loop antenna during uniaxial and biaxial stretching from 0 to 20%. S11 parameters of the loop antenna under strains along (**a**) *x*, (**b**) *y*, and (**c**) biaxial directions. Impedance of the loop antenna under strains along (**d**) *x*, (**e**) *y*, and (**f**) biaxial directions.

SAR Simulation

The simulation of SAR is conducted using HFSS and a standard human body model that includes the major body organs and structures. To simplify the simulation, the model contains only three layers: skin, fat, and muscle. Calculation involves a panel antenna with a gain of 11 dBi and a supply power of 15 W placed 1 m away from the closed point of the human body model. The thickness of individual layers varies according to different body locations. The focus is on three representative locations indicated by L1 to L3 are (Figure 3d). The simulation of SAR underneath the epidermal loop antenna (on a PDMS substrate with 20 μm thickness) uses planar layers of skin (2 mm in thickness), fat (2 mm), and muscle (10 mm) (Figure S5). The power available at the loop antenna is calculated based on Friis transmission, as described in the supplemental materials, and is 34 mW (at 1.16 GHz), with only 1.89 mW of reflected power.

REFERENCES

1 Loo Y-L, Someya T, Baldwin KW *et al.* Soft, conformable electrical contacts for organic semiconductors: High-resolution plastic circuits by lamination. *Proceedings of the National Academy of Sciences of the United States of America* 2002; **99**: 10252–10256.

2 Zaumseil J, Baldwin KW, Rogers JA. Contact resistance in organic transistors that use source and drain electrodes formed by soft contact lamination. *Journal of Applied Physics* 2003; **93**: 6117–6124.

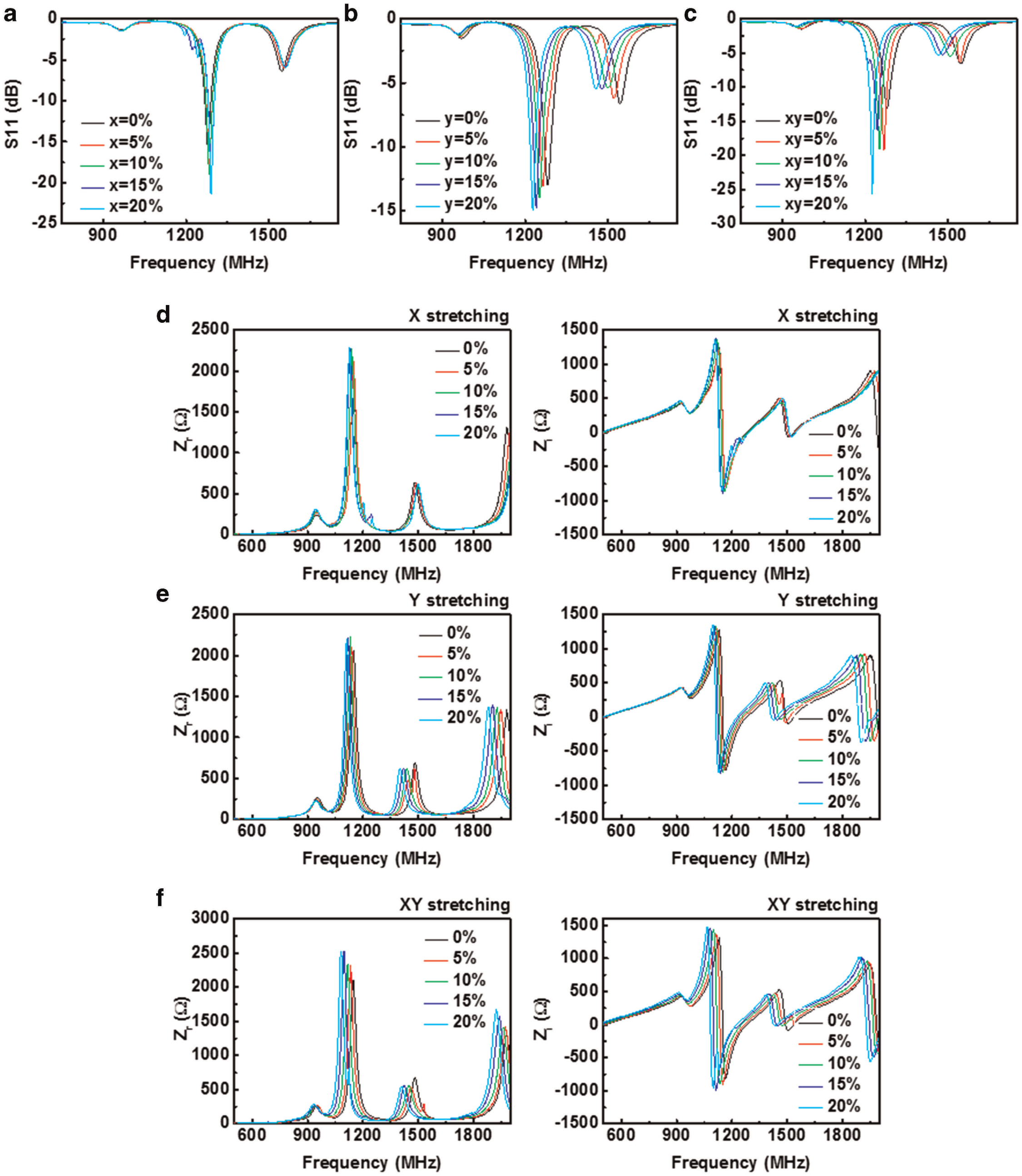

Figure S8 S-parameter measurement and impedance simulation of a dipole antenna during uniaxial and biaxial stretching from 0 to 20%. S11 parameters of the dipole antenna under strains along (**a**) *x*, (**b**) *y*, and (**c**) biaxial directions. Impedance of the dipole antenna under strains along (**d**) *x*, (**e**) *y*, and (**f**) biaxial directions.

3 Kim JB, Lee S, Toney MF *et al.* Reversible soft-contact lamination and delamination for non-invasive fabrication and characterization of bulk-heterojunction and bilayer organic solar cells. *Chemistry of Materials* 2010; **22**: 4931–4938.

4 Lee S, Ha J, Jo S *et al.* LEGO-like assembly of peelable, deformable components for integrated devices. *NPG Asia Materials* 2013; **5**: e66.

5 Huang J, Li G, Yang Y. A Semi-transparent plastic solar cell fabricated by a lamination process. *Advanced Materials* 2008; **20**: 415–419.

6 Hu L, Wu H, La Mantia F *et al.* Thin, flexible secondary li-ion paper batteries. *ACS Nano* 2010; **4**: 5843–5848.

7 Granstrom M, Petritsch K, Arias AC *et al.* Laminated fabrication of polymeric photovoltaic diodes. *Nature* 1998; **395**: 257–260.

8 Chabinyc ML, Salleo A, Wu Y *et al.* Lamination method for the study of interfaces in polymeric thin film transistors. *Journal of the American Chemical Society* 2004; **126**: 13928–13929.

9 Jacobs HO, Whitesides GM. Submicrometer patterning of charge in thin-film electrets. *Science* 2001; **291**: 1763–1766.

10 Ferguson G, Sigal G, Whitesides G *et al.* Contact adhesion of thin gold films on elastomeric supports- Cold welding under ambient conditions. *Science* 1991; **253**: 776–778.

11 Lu Y, Huang JY, Wang C *et al.* Cold welding of ultrathin gold nanowires. *Nature Nanotechnology* 2010; **5**: 218–224.

12 Liu C, Li Y-J, Sun S-G *et al.* Room-temperature cold-welding of gold nanoparticles for enhancing the electrooxidation of carbon monoxide. *Chemical Communications* 2011; **47**: 4481–4483.

13 Carlson A, Bowen AM, Huang Y *et al.* Transfer printing techniques for materials assembly and micro/nanodevice fabrication. *Advanced Materials* 2012; **24**: 5284–5318.

14 Kim C, Burrows PE, Forrest SR. Micropatterning of organic electronic devices by cold-welding. *Science* 2000; **288**: 831–833.

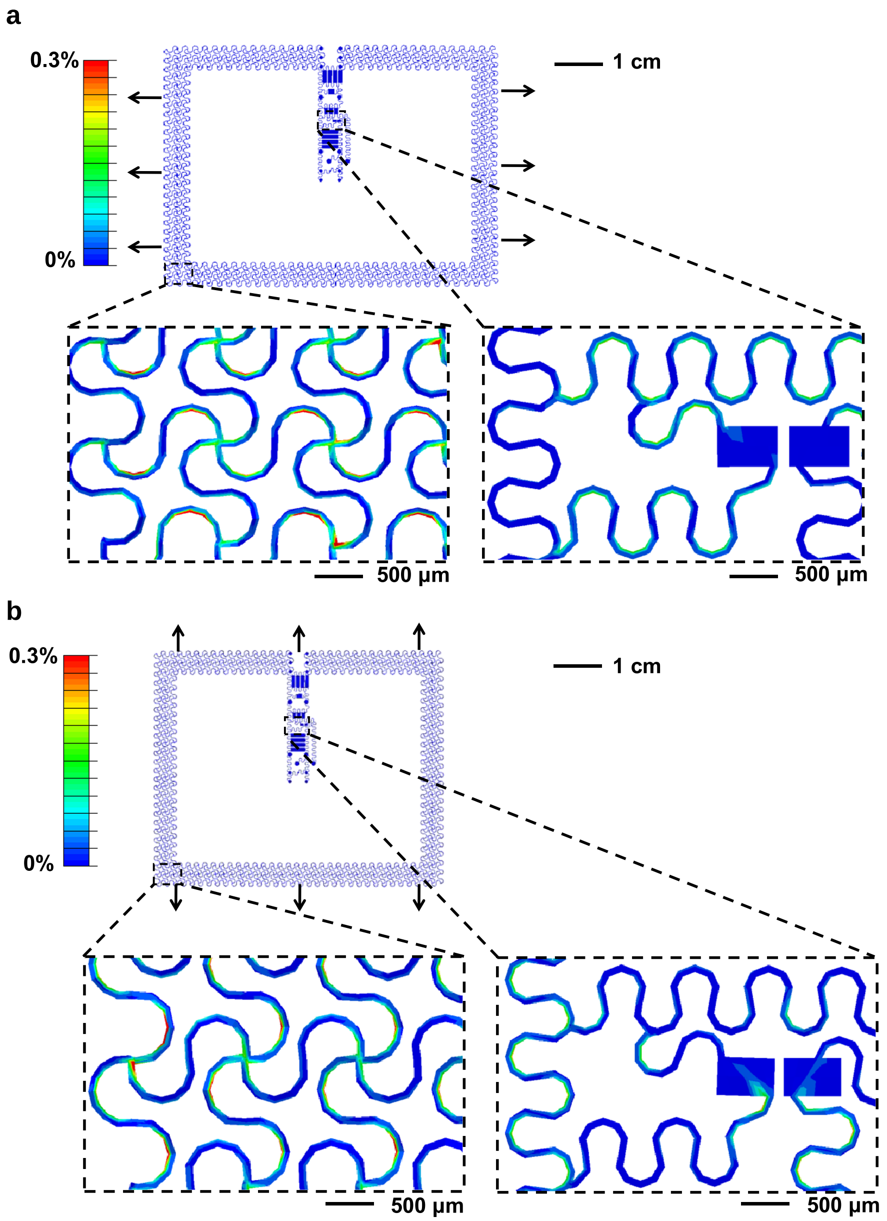

Figure S9 Mechanical simulation and analysis of an RF system assuming ~ 0.3% yield strain for copper. (**a**) A simulation result of the RF system under elastic stretching in the *x*-direction at ~ 6%. (**b**) A simulation result of the RF system under elastic stretching in the *y*-direction at ~ 6%.

15 Pereira ZS, da Silva EZ. Cold welding of gold and silver nanowires: a molecular dynamics study. *The Journal of Physical Chemistry C* 2011; **115**: 22870–22876.

16 Akande WO, Cao Y, Yao N *et al.* Adhesion and the cold welding of gold-silver thin films. *Journal of Applied Physics* 2010; **107** 043519-043519-8.

17 Wang S, Li M, Wu J *et al.* Mechanics of epidermal electronics. *Journal of Applied Mechanics* 2012; **79**: 031022–031022.

18 Simulia Abaqus 6.14 Documentation 2014. http://abaqus.software.polimi.it/v6.14/index.html.

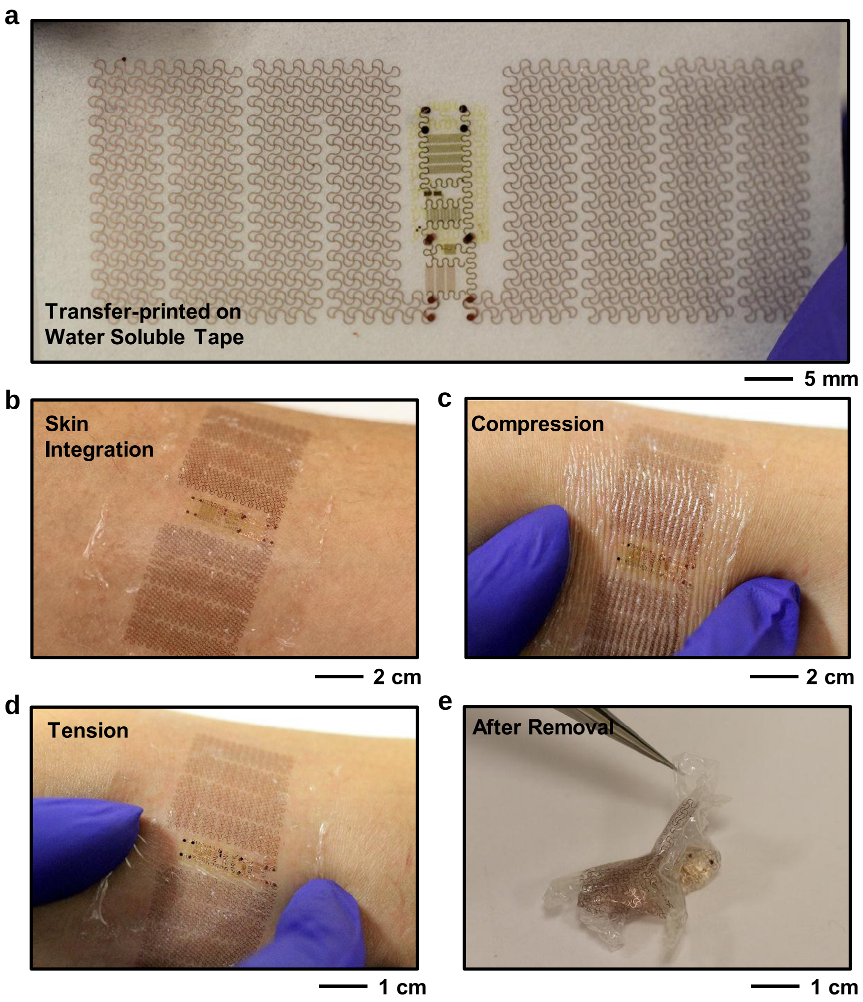

Figure S10 RF system with dipole antenna design. (**a**) A fully-integrated RF system with a dipole antenna, a voltage doubler, and an impedance matcher. (**b**) A RF system mounted on skin. (**c**) A RF system on skin under compressive stress. (**d**) A RF system on skin under tensile stress. (**e**)A RF system after peeling off from skin.

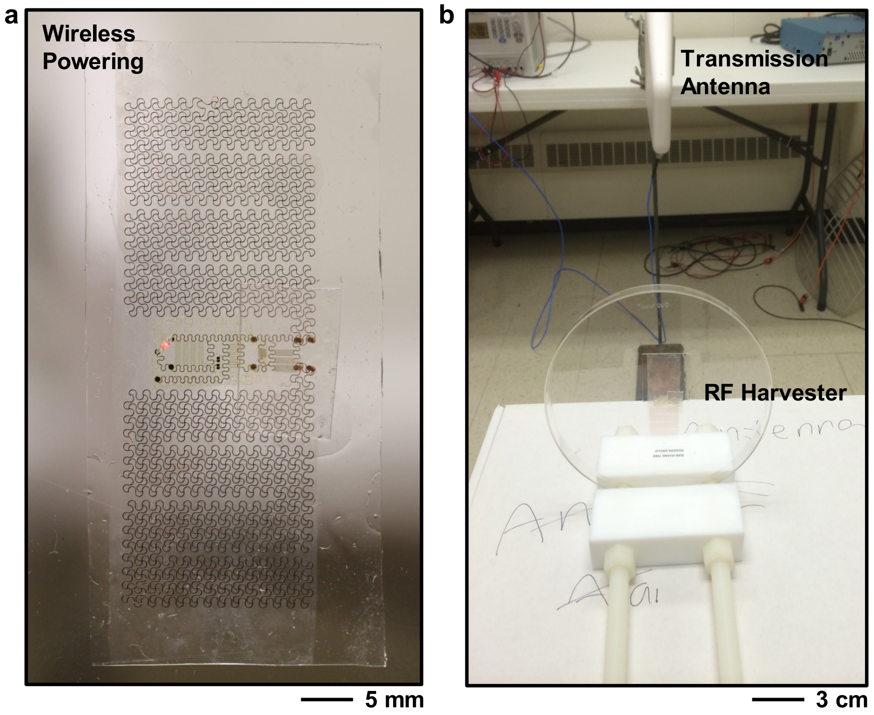

Figure S11 Wireless power harvesting demonstration. (**a**) Wirelessly-powered LED in a RF system with a dipole antenna. (**b**) Experiment setup with a RF transmission antenna as the source 1 meter away from the RF system.

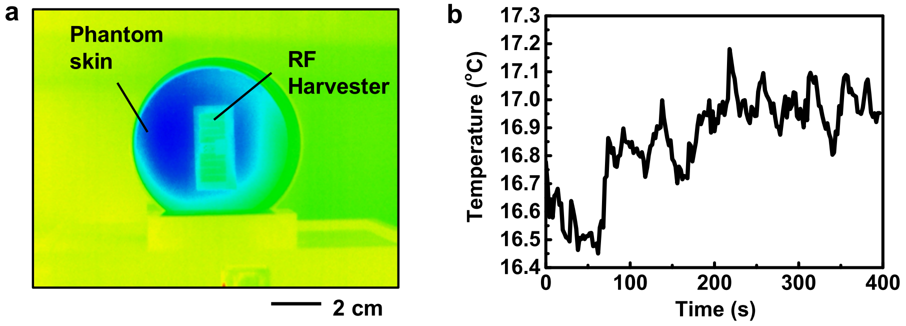

Figure S12 Temperature monitoring of a RF system during operation. (**a**) Infra-red (IR) thermograph of a RF system placed on a phantom skin substrate during exposure to RF power from a source (15 W, 1 GHz) located at a distance of 1 meter. (**b**) Temperature measured by the IR camera over a period of 400 s of continuous operation.

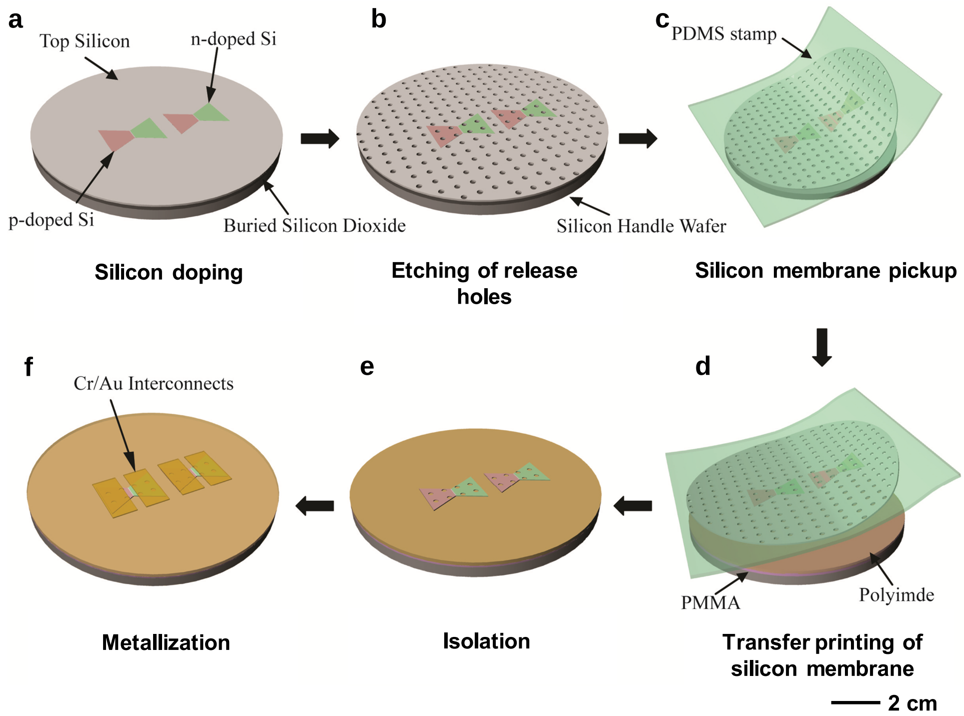

Figure S13 A fabrication process for the voltage doubler. (**a**) Diffusion doping of P and N regions on SiNM. (**b**) Formation of release holes by etching with RIE. (**c**) Release in HF and retrieval of the SiNM with a PDMS stamp. (**d**) Transfer printing onto a new substrate coated with a bilayer of PMMA/PI. (**e**) Isolation of PIN diodes by etching the remaining Si with RIE. (**f**) Deposition of Cr/Au interconnects.
